# Investigation of a Ventrodorsal Hippocampal Pathway to Regulate Cognition

**DOI:** 10.1016/j.bpsgos.2021.06.012

**Published:** 2021-08-16

**Authors:** Daniel J. Lodge

**Affiliations:** Department of Pharmacology and Center for Biomedical Neuroscience, University of Texas Health Science Center, and the South Texas Veterans Health Care System, Audie L. Murphy Division, San Antonio, Texas

SEE CORRESPONDING ARTICLE ON PAGE 101

Schizophrenia is a devastating psychiatric condition affecting approximately 1% of the population. While aberrant dopamine system function has long been associated with psychosis, the mechanisms contributing to cognitive and negative symptoms have not been conclusively elucidated. Alterations in hippocampal structure and function are consistently observed in patients with schizophrenia. Specifically, imaging studies have demonstrated hyperactivity at rest in the hippocampus, which is correlated with both psychosis (1) and cognitive dysfunction (2). Hippocampal hyperactivity can be modeled in rodents and has been associated with a decrease in the function of specific interneurons (those containing parvalbumin and somatostatin), which are also known to be altered in humans (3,4). This has led to the hypothesis that the hippocampus may be a novel site for therapeutic intervention in schizophrenia, with approaches aimed at reducing hippocampal hyperactivity (5).

The hippocampus is a temporal lobe structure historically associated with the consolidation of memory. Based on anatomical connectivity and gene expression analyses, there is increasing evidence that the hippocampus possesses functionally distinct subregions along its dorsal/ventral axis (the anterior/posterior axis in humans) (6). Thus, the dorsal hippocampus receives multimodal sensory information from cortical regions and plays a key role in cognition. In contrast, the ventral hippocampus is connected more intimately with limbic structures related to stress, emotion, and affect. In patients with schizophrenia, baseline hippocampal hyperactivity is more prominent in anterior regions, consistent with data from rodent models showing hyperactivity within the ventral areas. Moreover, research in rodent models suggests that ventral hippocampal hyperactivity contributes to augmented dopamine system function via projections to the nucleus accumbens, whereas aberrant ventral hippocampal drive of the prefrontal cortex may contribute to negative and cognitive symptoms (7).

It should be noted that functionally discrete subregions of the hippocampus are extensively interconnected to form intrahippocampal circuits. As initially proposed by Ramón y Cajal, the most well-characterized route of information flow in the hippocampus is through the trisynaptic pathway. In this glutamatergic circuit, projections from the entorhinal cortex innervate granule cells of the dentate gyrus (DG), whose mossy fiber projections excite CA3 pyramidal neurons that send projections, via the Schaffer collateral pathway, to the CA1 region. However, many studies have highlighted the importance of nontrisynaptic components of hippocampal circuitry that appear critical for regulating cognitive function. Moreover, within hippocampal circuits, alterations in ventral regions may influence dorsal circuits and vice versa. Indeed, this is the premise of an elegant and impactful article by Bauer *et al.* (8) in the current issue of *Biological Psychiatry: Global Open Science* that examines the hypothesis that hyperactivity of mossy cells in the ventral DG results in activation of the dorsal DG and alterations in cognitive function. To model the functional consequences of hyperactivity within the long-range projections from the ventral hippocampus, the authors focused on mossy cells located within the ventral DG that project across the longitudinal axis of the hippocampus. These glutamatergic neurons synapse within the dorsal hippocampal DG, where they target both granule cells and interneurons. Previous studies have shown that optogenetic or chemogenetic manipulation of mossy cells in the ventral DG can impair dorsal hippocampal–dependent spatial memory, suggesting critical interactions between the ventral and dorsal regions (9,10).

To examine the activity of ventral mossy cells during exploration, Bauer *et al.* (8) performed in vivo fiber photometry in freely behaving mice to record calcium transients using the genetically encoded calcium indicator GCaMP6f. Selectivity for the ventral hippocampal mossy cells projecting to the dorsal DG was obtained by a combination viral approach, whereby Cre recombinase was expressed in neurons projecting to the dorsal DG by administration of a retrograde adeno-associated virus. Selectivity for ventral DG–dorsal DG projections was then obtained by Cre-dependent expression of GCaMP6f within the ventral DG. This technique was validated by immunohistochemistry, using calretinin as a marker, with ∼90% of the GCaMP6f-positive neurons being DG mossy cells. Using this sophisticated approach, the activity of ventral DG–dorsal DG mossy cells was examined in response to exploratory information gathering in an open field arena. Interestingly, robust activation of ventral mossy cells was observed during rearing events, when a mouse changes its vantage point to integrate multisensory cues from the environment, but not during horizontal exploration. To examine the consequence of ventral DG hyperactivation on dorsal hippocampal–dependent behaviors, a G_q_-coupled DREADD (designer receptor exclusively activated by designer drugs) was expressed bilaterally in ventral mossy cells and activated by the systemic administration of clozapine N-oxide. These data show that chemogenetic activation of ventral mossy cells during the training phase of an object location memory task resulted in impaired performance when examined 24 hours later. Interestingly, previous work examining the role of mossy cells in the context of epilepsy (9) found that optogenetic inhibition of ventral mossy cells during the learning phase of the object location memory task resulted in an impairment, suggesting that activity is necessary during spatial memory encoding. Together, these data show that mossy cell activity in healthy mice is tightly regulated such that either hyperactivation or inhibition can induce deficits in cognitive function.

These studies complement recent work (10) showing that activity of ventral mossy cells correlates with environmental novelty and that manipulation of ventral mossy cell activity bidirectionally regulates novelty-induced contextual memory acquisition. Taken together, the article by Bauer *et al.* (8) advances our understanding of the role of intrahippocampal circuits contributing to cognition. Thus, mossy cells of the ventral DG are activated in a novel environment during information gathering, and alterations in their activity lead to functional effects on dorsal hippocampal–dependent cognitive function. Furthermore, while evidence suggests that ventral and dorsal hippocampal subdivisions are functionally distinct (6), these studies indicate that information can be relayed through a ventrodorsal hippocampal pathway to regulate cognition. Such information provides the foundation for future studies examining whether mossy fiber cells of the ventral DG are altered in models of chronic hippocampal hyperactivity and whether manipulation of these neurons may reverse cognitive deficits observed in rodent models used to study circuit-based alterations associated with schizophrenia.
